# Inverse design of photonic and phononic topological insulators: a review

**DOI:** 10.1515/nanoph-2022-0309

**Published:** 2022-08-22

**Authors:** Yafeng Chen, Zhihao Lan, Zhongqing Su, Jie Zhu

**Affiliations:** State Key Laboratory of Advanced Design and Manufacturing for Vehicle Body, Hunan University, Changsha, Hunan 410082, P.R. China; Department of Mechanical Engineering, Hong Kong Polytechnic University, Hong Kong SAR, P.R. China; Department of Electronic and Electrical Engineering, University College London, London WC1E 7JE, UK; School of Physics Science and Engineering, Tongji University, Shanghai 200092, P.R. China

**Keywords:** inverse design, phononic crystals, photonic crystals, topological insulators

## Abstract

Photonic and phononic topological insulators (TIs) offer numerous opportunities for manipulating light and sound with high efficiency and resiliency. On the other hand, inverse design methodologies, such as gradient-based approaches, evolutionary approaches, and deep-learning methods, provide a cost-effective strategy for developing photonic and phononic structures with unique features in steering light and sound. Here, we discuss recent advances and achievements in the development of photonic and phononic TIs employing inverse design methodologies, including one-dimensional TIs, TIs based on the quantum spin Hall effect (QSHE) and quantum valley Hall effect (QVHE), and high-order TIs in lattices with diverse symmetries. Several inversely designed photonic and phononic TIs with superior performance are exhibited. In addition, we offer our perspectives on the future of this emerging study field.

## 1 Introduction

The discovery of topological insulators (TIs) opens a new chapter in condensed matter physics. A key feature of TIs is the existence of topologically protected edge states at the interface between two materials with distinct topological invariants, which are immune to backscattering and robust against impurities and defects, providing possibilities for developing novel topological devices [1, 2]. Subsequently, the concept of TIs has been applied to the photonic and phononic systems. Several variants of photonic and phononic TIs based on distinct physical mechanisms, such as quantum Hall effect (QHE) [3–5], quantum spin Hall effect (QSHE) [6–10], and quantum valley Hall effect (QVHE) [11–17], have been proposed and implemented to mimic TIs of condensed matter physics. These photonic and phononic TIs adhere to conventional bulk-boundary correspondence and support gapless edge states, that is, *n*-dimensional (*n*D) TIs support (*n*-1)D edge states. Most recently, a new kind of TIs, also called the high-order TIs, has been proposed [18]. Going beyond the conventional bulk-boundary correspondence, an *n*D *m*th-order TI hosts (*n* − 1)D, (*n* − 2)D, …, (*n* − *m* + 1)D gapped edge states and (*n* − *m*)D gapless edge states. For example, in 2D systems, second-order TIs support gapped 1D edge states and 0D in-gap corner states. Meanwhile, the concept of high-order TIs has been rapidly expanded into photonic and phononic systems. So far, several kinds of second-order and third-order photonic and phononic TIs have been realized. In 2D systems, crystalline symmetry determines the underlying physics of second-order photonic and phononic TIs. Hitherto, various second-order photonic and phononic TIs have been created in lattices with different symmetries, such as C_3_ symmetric lattice [19–21], C_6v_ symmetric hexagonal lattice [22–24], and C_4_ and C_4v_ symmetric square lattice [25–29]. The realization of these conventional and high-order photonic and phononic TIs has overturned some of the traditional views of light and sound propagations and created unprecedented opportunities for steering light and sound with high efficiency and robustness. Readers can refer to recent reviews [18, 30], [31], [32], [33], [34], [35] for the development of photonic and phononic TIs.

The performance of photonic and phononic TIs highly depends on the configurations of photonic and phononic structures. However, prevailing photonic and phononic TIs are mainly designed by empirical methods and are generally made of regular photonic and phononic structures, whose geometry parameters are determined via trial and error. Hence, the performance of the designed photonic and phononic TIs may be severely limited. For example, the operation bandwidth of topological edge states is narrow and the quality factor of topological corner states is small. One potential solution to these problems is to employ inverse design approaches, which are algorithmic techniques for finding the optimal material layout to achieve the best objective performance, to design photonic and phononic TIs. The commonly used inverse design techniques include gradient-based approaches (such as topology optimization method or adjoint method) [36, 37], evolutionary approaches (such as genetic algorithms or particle swarm algorithms) [38, 39], and deep-learning methods [40]. The gradient-based approaches need to calculate derivative information of the objective function about the individual design parameter, that is, the sensitivity of each element. Once the sensitivities are obtained, the optimal solution can be quickly obtained after several iterations. However, for some physics scenarios, where the sensitivity is either not easily accessible or unreliable, evolutionary approaches and deep-learning methods are favored. Nonetheless, compared to the gradient-based approaches, they tend to be orders of magnitude more computationally expensive as they need a large database to update the solution or train the model. Over the past few years, inverse design techniques have been widely utilized in designing novel photonic and phononic structures that surpass empirically designed structures in many applications, such as photonic and phononic crystals [41–52], metamaterials [53–59], metasurfaces, and metastructures [37, 60], [61], [62], [63], [64], [65], [66], [67], [68], [69], [70], [71], [72]. Readers can refer to recent reviews [73–81] for the development of this topic.

Recently, researchers began to exploit inverse design techniques to design photonic and phononic TIs to enhance their performances. In this review, we intend to summarize the developments of this emerging field that combines photonic and phononic TIs with inverse design techniques and offer perspectives on the future development directions in this field. This review is organized as follows: Section 2 provides an overview of works on the inverse design of photonic and phononic TIs following conventional bulk-boundary correspondence, including 1D photonic TIs and 2D photonic and phononic TIs based on QSHE and QVHE. Section 3 surveys works on the inverse design of high-order photonic and phononic TIs in lattices with different symmetries, including C_4v_, C_3_, and C_6v_ symmetries. Section 4 summarizes the work and gives some prospects on the future development directions for this research field.

## 2 Inverse design of photonic and phononic TIs following conventional bulk-boundary correspondence

Currently, works on inverse design of photonic and phononic TIs following conventional bulk-boundary correspondence mainly focus on 1D and 2D systems, which will be reviewed in Sections 2.1 and 2.2, respectively.

### 2.1 Inverse design of 1D photonic and phononic TIs via deep learning

To date, the intelligent design approaches adopted for designing 1D topological systems are mainly based on deep learning, including forward prediction and inverse design. The forward prediction method is used to identify the topology properties of the given structure, whereas the inverse design method is adopted to search the ideal structure that satisfies the desired topology properties. In the forward prediction model for designing topological structures, the input and output have a one-to-one relationship; thus, it is effective to train the deep learning model to accurately predict the topology properties of the given structures. Up to now, various forward prediction models based on deep learning have been successfully exploited to identify topology properties (Chern number, winding number, and Zak phase) of systems in condensed matter physics and photonics [82–91]. Here, we mainly focus on works using deep learning to inversely design topological structures. In 1D photonic topological systems, the topological property of each band is usually characterized by Zak phase, defined by [92]

(1) $$\theta_{n}=\overset{\pi /a}{\underset{-\pi /a}{\int }}\left(i\underset{\text{cell}}{\int }dx\varepsilon \left(x\right)u_{n,k}^{*}\left(x\right)\partial_{k}u_{n,k}\left(x\right)\right)\text{d}k$$

where *u*_*n*,*k*_ denotes the eigenstate of the *n*th band and $\varepsilon \left(x\right)$ denotes the dielectric constant at position *x*. The 1D system with inversion symmetry always has two inversion centers and the Zak phase is quantized at either 0 or *π* if the origin is chosen to be one of the inversion centers [92].

**Figure 1:**
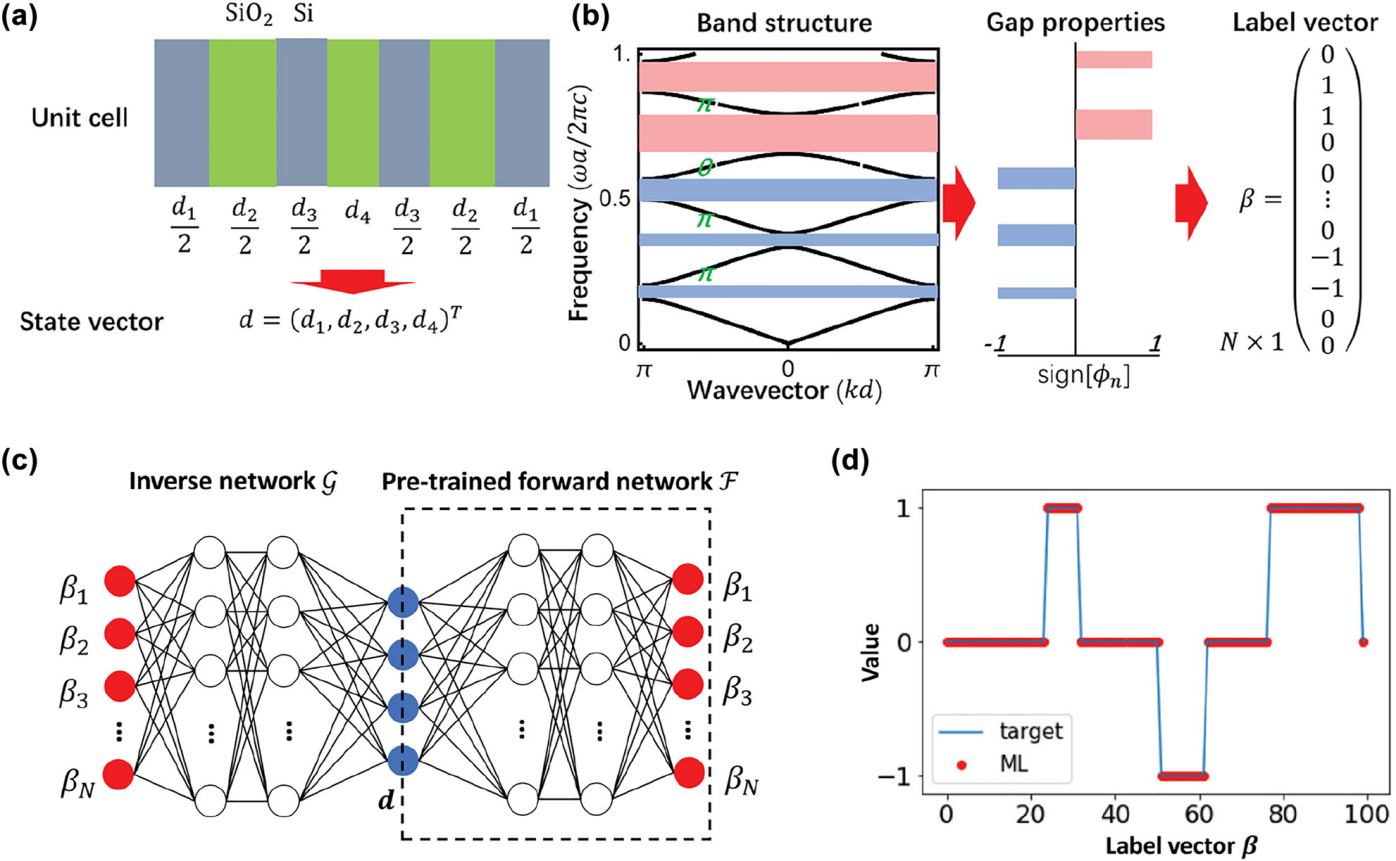
Inverse design of 1D photonic topological insulators using deep learning [93]. (a) The schematic of the state vector consisting of geometry parameters. (b) The schematic of label vector consisting of reflection phases. (c) The schematic of the tandem network. (d) The comparison between the reflection phases of the inverse-designed photonic crystal and the target reflection phases.

Recently, Long et al. [93] exploited deep learning techniques to design structures of 1D photonic crystals that satisfy the objective Zak phase properties. The unit cell of the 1D photonic crystal is composed of several layers of SiO_2_ and Si. The length of each layer of the dielectric media is represented by the state vector ***d*** = (*d*_1_, *d*_2_, …, *d*_*M*_)^*T*^ [Figure 1(a)], which denotes the geometry parameters of the photonic crystal. They used the reflection phases of the bandgaps, which are associated with the Zak phase of each band, to characterize the topology properties

(2) $$\sin\left(\phi_{n-1}\right)/\mathrm{sign}\left(\phi_{n}\right)=-{\text{e}}^{\text{i}\theta_{n}}$$

where *ϕ*_*n*_ = (−*π*, *π*) denotes the reflection phase of the *n*th bandgap. According to Eq. (2), the Zak phase of each band can be derived once the reflection phase of each bandgap is known. The frequency region of interest [*ω*_min_, *ω*_max_] is divided into *N* parts *ω*_*j*_, *j* = 1, …, *N*. A label vector ***β***, an *N* × 1 vector, consisting of the reflection phase properties for each frequency point *ω*_*j*_ is introduced: *β*_*j*_ = sign(*ϕ*_*n*_) for the bandgap; otherwise, *β*_*j*_ = 0 [Figure 1(b)]. With the state vector ***d*** and label vector ***β***, the forward prediction model, ***β*** = $F\left(d\right)$ , and the inverse design model, ***d*** = $G\left(\beta \right)$ , are constructed. As one photonic structure has only one form of band dispersion (one-to-one), training the forward prediction model converges quickly. However, for training the inverse design model, as one form of band structure will not correspond to only one case of the photonic structure (one-to-many), it converges slow or even diverges. To overcome this problem, they built a tandem network consisting of the inverse network followed by a pretrained forward network (Figure 1(c)), which resembles an autoencoder, where G encodes the label vector into the state vector, and then, this state vector is decoded into the original label by F . Thereafter, the tandem network is trained by minimizing the loss between the label vectors and the predicted ones, $\min\frac{1}{L}\underset{i}{\sum }{\left|F\left(G\left(\beta_{i}\right)\right)-\beta_{i}\right|}^{2}$ , to find the appropriate G . The tandem network converges after about 5000 epochs. As can be seen in Figure 1(d), the network is able to find the photonic crystal that satisfies the target label vectors, validating the effectiveness of the proposed method. Adopting a similar tandem network strategy, Singh et al. [94] designed a 1D photonic crystal having target topological band structures. Apart from using the tandem network strategy to handle the one-to-many problem, Pilozzi et al. [95] introduced categorical features that specify the domains of frequency and eigenmode as additional input variables to design the 1D Aubry–Andre–Harper topological insulator with topological edge states at target frequencies. To the best of our knowledge, using inverse design methods to design 1D phononic topological insulators has not been reported yet.

### 2.2 Inverse design of 2D photonic and phononic TIs

The quantum Hall versions of photonic and phononic TIs need external fields, such as external magnetic fields and circular flow, to break the time-reversal symmetry, making them difficult to be implemented. To overcome this problem, photonic and phononic TIs based on QSHE and QVHE are proposed, which are made of purely passive materials preserving the time-reversal symmetry. Meanwhile, inverse design methods have been exploited to devise them to widen the operation bandwidth of topological edge states. In this section, we will review works on using inverse design methods to construct photonic and phononic TIs based on QSHE and QVHE, respectively.

#### 2.2.1 Inverse design of QSHE-based photonic and phononic TIs

The physics-inspired method to design QSHE-based photonic and phononic TIs generally includes three steps: designing a structure with a double Dirac cone degenerated by two dipolar (D) modes *p*_*x*_/*p*_*y*_ and two quadrupolar (Q) modes $d_{x^{2}-y^{2}}/d_{xy}$ ; shrinking (expanding) the lattice sites to open the double Dirac cone with the two Q modes above (below) the two D modes to design a trivial (nontrivial) structure; and combining the trivial and nontrivial structures to form an interface to support pseudospin-up and pseudospin-down topological edge states. In order to obtain a large overlapped bandgap between trivial and nontrivial structures to enable topological edge states with a wide bandwidth, several inverse design strategies have been proposed. Firstly, we will review different inverse design strategies for designing photonic and phononic TIs in hexagonal lattices, which will be presented according to the optimization scheme and the time sequence. Then, we will present inverse design strategies for designing QSHE-based sonic and phononic TIs in square lattices in a similar way.

**Figure 2:**
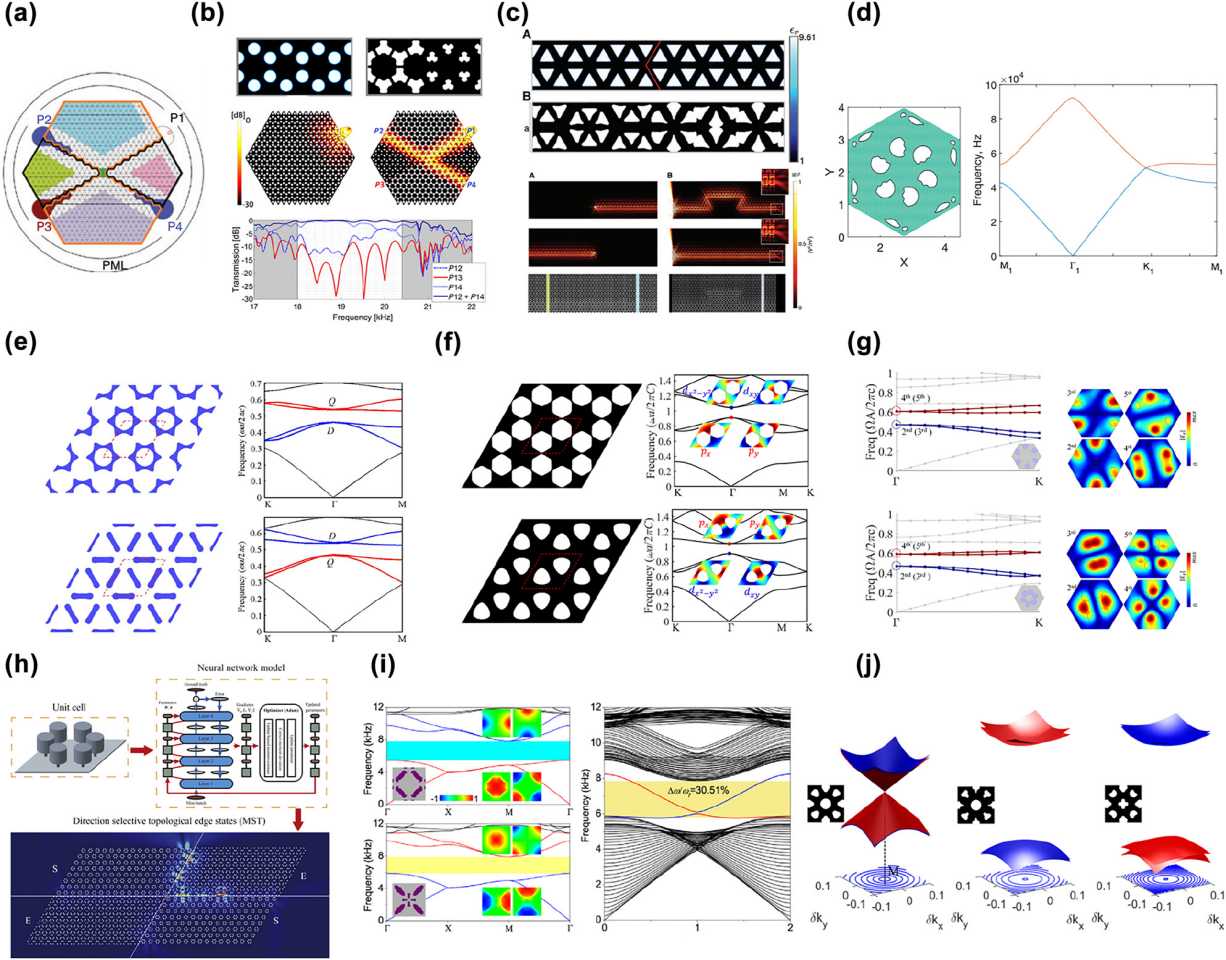
Inverse-designed photonic and phononic TIs based on QSHE. (a) Schematic of the waveguide used for designing acoustic and photonic TIs in Refs. [96, 97]. (b) Optimized acoustic TI in Ref. [96]. (c) Optimized photonic TI in Ref. [97]. (d) Optimized phononic crystal with a Dirac cone [98]. (e) Optimized trivial and nontrivial photonic crystals designed by maximizing LDOSs [100]. (f) Optimized trivial and nontrivial acoustic crystals designed by maximizing powers emitted by artificially assigned sources [101]. (g) Optimized nontrivial and trivial photonic crystals by the MMCs-based inverse design method [102]. (h) Schematic of the inverse design strategy based on machine learning for designing topological metaplates [103]. (i) Optimized nontrivial and trivial sonic crystals in square lattices [104]. (j) Optimized nontrivial and trivial phononic crystals in square lattices [105].

Based on the transmission property of QSHE-based topological edge states, Christiansen et al. [96, 97] developed a topology optimization framework to synthesize QSHE-based acoustic and photonic TIs by engineering transmissions of the different ports in a well-arranged waveguide (Figure 2(a)). In the waveguide, the upper and lower colorful domains were made of one kind of unit cells, whereas the right and left colorful domains were made of the other kind of unit cells; four channels were formed between these domains. According to the spin-locked property of the topological edge states in QSHE-based systems, waves launched from port P1 could only transport to ports P2 and P4 but could not propagate to port P3. Based on this unique property, the optimization problem was formulated as maximizing field intensity magnitudes at ports P2 and P4 and simultaneously minimizing the field intensity magnitude at port P3. Figure 2(b) and (c) show the optimized acoustic TI with a relative bandwidth of 12.5% [96] and photonic TI with the relative bandwidth of 6% [97], respectively. However, this method needs to optimize the waveguide consisting of a large number of unit cells, it consumes significant computation resources. It is more efficient to design the trivial and nontrivial unit cells directly based on their physical properties.

As trivial and nontrivial unit cells in QSHE-based TIs are evolved from the unit cell with double Dirac cone. Nanthakumar et al. [98] used a level set-based topology optimization approach [99], a steepest descent method by combining the shape sensitivity analysis with the Hamilton–Jacobi equation for moving the level-set function, to create a Dirac cone with two-fold degeneracy at the *K* point of the phononic crystal for the out-of-plane modes (Figure 2(d)), based on which a double Dirac cone at the Γ point was created using the zone folding mechanism, and thereafter, trivial and nontrivial unit cells were designed by manually adding or removing materials at the center or at the six corners of the hexagonal unit cell to lift the degeneracy of the double Dirac cone. Topological edge states with a bandwidth of 4.08% were formed at the interface between the trivial and nontrivial structures. As the trivial and nontrivial unit cells were still designed by trial and error, the bandwidth of topological edge states is narrow. To overcome this problem, Chen et al. [100] developed a topology optimization method to accurately control the frequencies of D and Q modes to design the nontrivial and trivial photonic crystals. The D (Q) modes could be excited at the target frequency *ω*_D_ (*ω*_Q_) by maximizing the local density of states (LDOSs) emitted by a source at specific position with the frequency of *ω*_D_ (*ω*_Q_). The trivial (nontrivial) photonic crystals could be obtained by setting *ω*_D_ < *ω*_Q_ (*ω*_D_ > *ω*_Q_) and maximizing the corresponding two LDOSs simultaneously (Figure 2(e)). By gradually enlarging the difference between *ω*_D_ and *ω*_Q_, large overlapped bandgap between trivial and nontrivial photonic crystals could be created. In doing so, topological edge states with a record-breaking bandwidth of 14% (relative size) were reported. Moreover, as the frequencies of *D* and *Q* modes could be set as desired values, the frequency of the topological edge state could be controlled on-demand when the lattice size is fixed, providing more flexibility in practical applications. Thereafter, Chen et al. [101] extended this method to the design of acoustic TIs based on QSHE. The *D* (*Q*) modes were excited at the target frequency by maximizing the power emitted by an artificially assigned source. Trivial and nontrivial sonic crystals with wide overlapped bandgap were designed (Figure 2(f)). Topological edge states with the bandwidth of 13.1% (relative size) were formed at the interface between the trivial and nontrivial sonic crystals, whose robustness against defects and disorders was further demonstrated. Luo et al. [102] developed a moving morphable components (MMCs)-based inverse design formulation for designing photonic TIs based on the QSHE. Unit cells with specific symmetry were described using MMCs via several design variables. Two constraint functions controlling the lifting of double Dirac cone and the band inversion were imposed. Through maximizing the working bandwidth of the topological edge band of the corresponding supercell, a pair of trivial and nontrivial photonic crystals was designed (Figure 2(g)). The relative size of the bandwidth of the topological edge band was 12.2%. TIs in Figure 2(a)–(g) are all designed using traditional topology optimization methods. He et al. [103] exploited the machine learning technique to inversely design QSHE-based topological metaplates for flexural waves (Figure 2(h)). The size and topological properties (trivial or nontrivial) of the bandgaps were controlled by the arrangement radius R of the resonators of the meta-plate. In the neural network, the bandgap width and 0/1 (0 denotes trivial unit cell and 1 denotes nontrivial unit cell) were set as the input, whereas the arrangement radius R was set as the output. After training, the neural network accurately predicted the values of R for the trivial and nontrivial unit cells with the target bandgap width.

The aforementioned works all focused on designing QSHE-based photonic and phononic TIs in hexagonal lattices. To design QSHE-based photonic and phononic TIs in square lattices, Dong et al. [104] adopted the genetic algorithm to design nontrivial and trivial sonic crystals in square lattices by maximizing the power emitted by the specific source to excite the *D* and *Q* modes at the target frequencies (Figure 2(i)). Topological edge states with a relative bandwidth of 30.51% were formed at the interface between trivial and nontrivial domains. Lu et al. [105] presented a level set-based computational methodology for the inverse design of QSHE-based phononic TIs in lattices with C_4(v)_ or C_2(v)_ symmetry for the out-of-plane modes. The modal assurance criterion (MAC), i.e., the square of normalized inner product of the displacement field, was introduced into the optimization formulation to induce the band inversion between *D* and *Q* modes. Trivial and nontrivial phononic crystals with an overlapped bandgap of a relative size of 8.65% were designed (Figure 2(j)).

#### 2.2.2 Inverse design of QVHE-based photonic and phononic TIs

The physics-inspired method to design QVHE-based TIs includes four steps [106]: in step 1, constructing a unit cell with C_3v_ symmetry to form a Dirac cone at the high symmetry points *K*/*K*′; in step 2, breaking the mirror or inversion symmetry of the base unit cell to gap the Dirac cone, inducing a pair of opposite valley Chern numbers at *K* and *K*′; in step 3, constructing an inversion-symmetric partner of the unit cell in step 2, resulting in the inversion of valley Chern numbers at *K* and *K*′; and in step 4, building an interface between the unit cells in step 2 and step 3 to host the topological edge states. However, the bandwidth of the obtained edge states based on this physics-inspired method was narrow.

**Figure 3:**
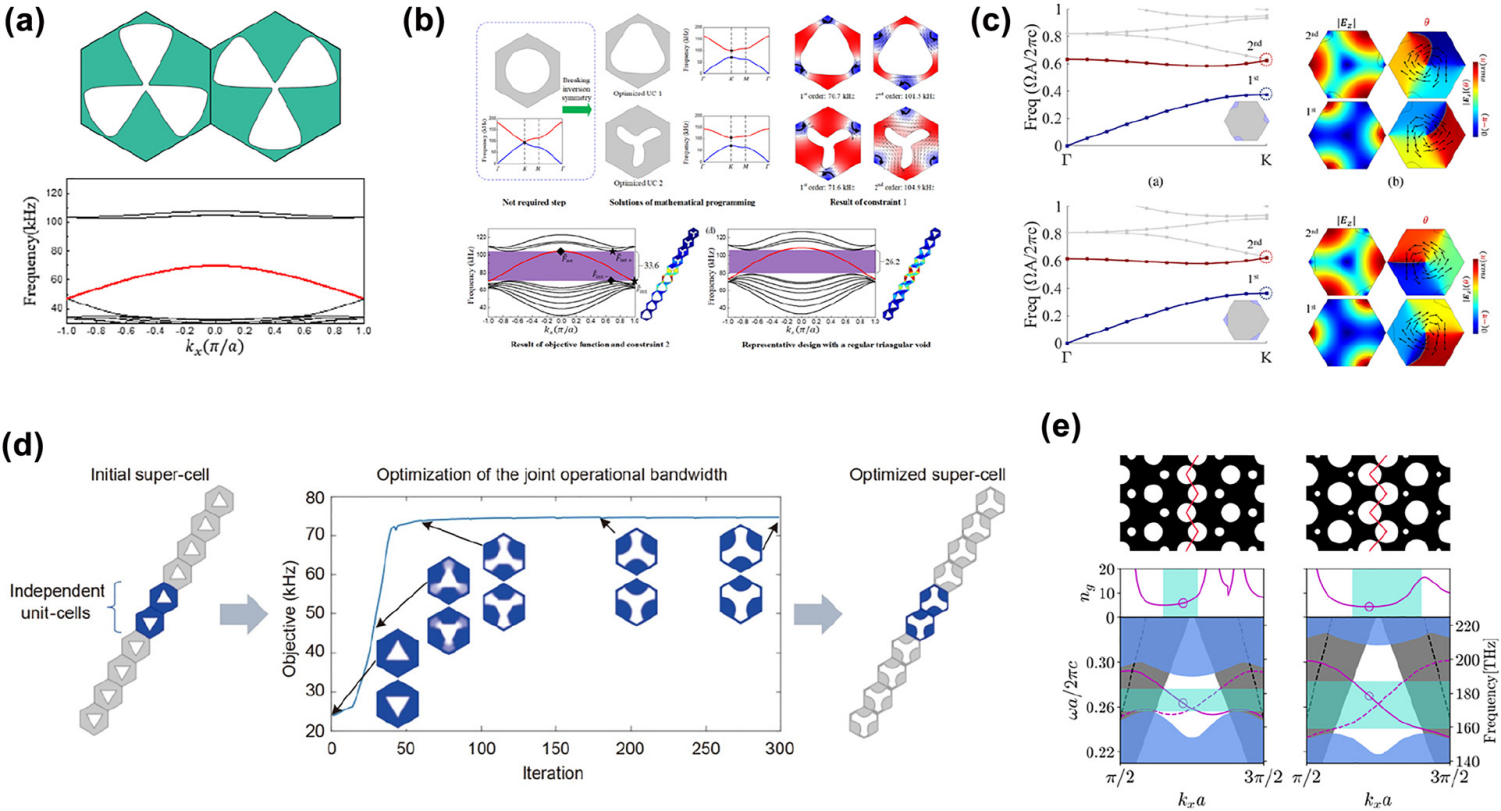
Inverse-designed photonic and phononic TIs based on QVHE. (a) Optimized unit cells for constructing QVHE-based phononic TIs using the first strategy in Ref. [107] and the dispersion diagram of the topological edge states. (b) Optimized unit cells using the second strategy in Ref. [107] and the dispersion diagram of the topological edge states. (c) Optimized unit cells for constructing QVHE-based photonic TIs in Ref. [102]. (d) Optimized supercells for constructing QVHE-based phononic TIs in Ref. [108]. (e) Initial (left panel) and optimized (right panel) QVHE-based topological photonic slabs in Ref. [109] and their dispersion diagrams of the corresponding topological edge states.

To enlarge the bandwidth of the edge states, Du et al. [107] developed two inverse design strategies for designing QVHE-based phononic TIs in 2D elastic systems, where the out-of-plane modes were considered. The first strategy was to maximize the common width of the first bandgap of a pair of unit cells; meanwhile a mode inversion error constraint was introduced to induce the band inversion. Figure 3(a) shows one pair of the optimized unit cells and the edge states formed between them. It can be seen that, even though a large common width was obtained, a wide edge-state gap appeared, because the peak value of Berry curvature decreases dramatically and the Berry curvature is unconfined in the reciprocal space as the width of common bandgap increases. It also means that the common bandgap width cannot accurately measure the operating bandwidth of the edge states; the edge-state gap could also diminish the topological robustness. To tackle this problem, a second strategy was proposed to directly maximize the bandwidth of edge states of the supercell consisting of two kinds of unit cells with a mode inversion error constraint. Figure 3(b) shows the optimized unit cells and the dispersion diagram of the edge states, from which it can be found that a wide edge band, with a relative size of 33.6% filling the whole bandgap, was obtained. Thereafter, Luo et al. [102] extended this strategy to design QVHE-based photonic TIs with a wide working bandwidth, whose relative size was reported to be 8.5% (Figure 3(c)). Likewise, Zhang et al. [108] designed a QVHE-based phononic TI supporting ultra-broadband edge-states by maximizing the operation bandwidth of the topological edge states of a supercell via topology optimization (Figure 3(d)). The operation bandwidth of the topological edge states for the optimized TI was 74.5 kHz ([46.4 kHz, 120.9 kHz]) with a relative size of 89.1%.

The above inverse-designed QVHE-based TIs are all ideal 2D systems. Nussbaum et al. [109] adopted an automatic differentiation-enabled inverse design strategy to design a QVHE-based topological photonic slab to enlarge the operation bandwidth. The optimization started from the initial structure introduced in Ref. (13] [left panel of Figure 3(e)). A two-step optimization strategy was adopted: first, the hole sizes in the photonic crystal that the waveguide is built from were optimized to increase the size of the bandgap; then the holes around the interface were optimized for enlarging the bandwidth of the topological edge states. After the optimization, as shown in the right panel of Figure 3(e), the normalized bandwidth of the topological edge states was enlarged from 7.5% to 16.2%.

## 3 Inverse design of high-order photonic and phononic TIs

Previous works on designing high-order photonic and phononic TIs all aimed at 2D systems, that is, designing second-order photonic and phononic TIs. Engineering lattice symmetries play a vital role in designing high-order photonic and phononic TIs. To date, inverse design strategies have been developed for designing second-order photonic and phononic TIs in lattices with different symmetries, including C_4v_, C_3_, and C_6_, which will be reviewed in the following subsections, respectively.

### 3.1 C_4v_-symmetric lattices

**Figure 4:**
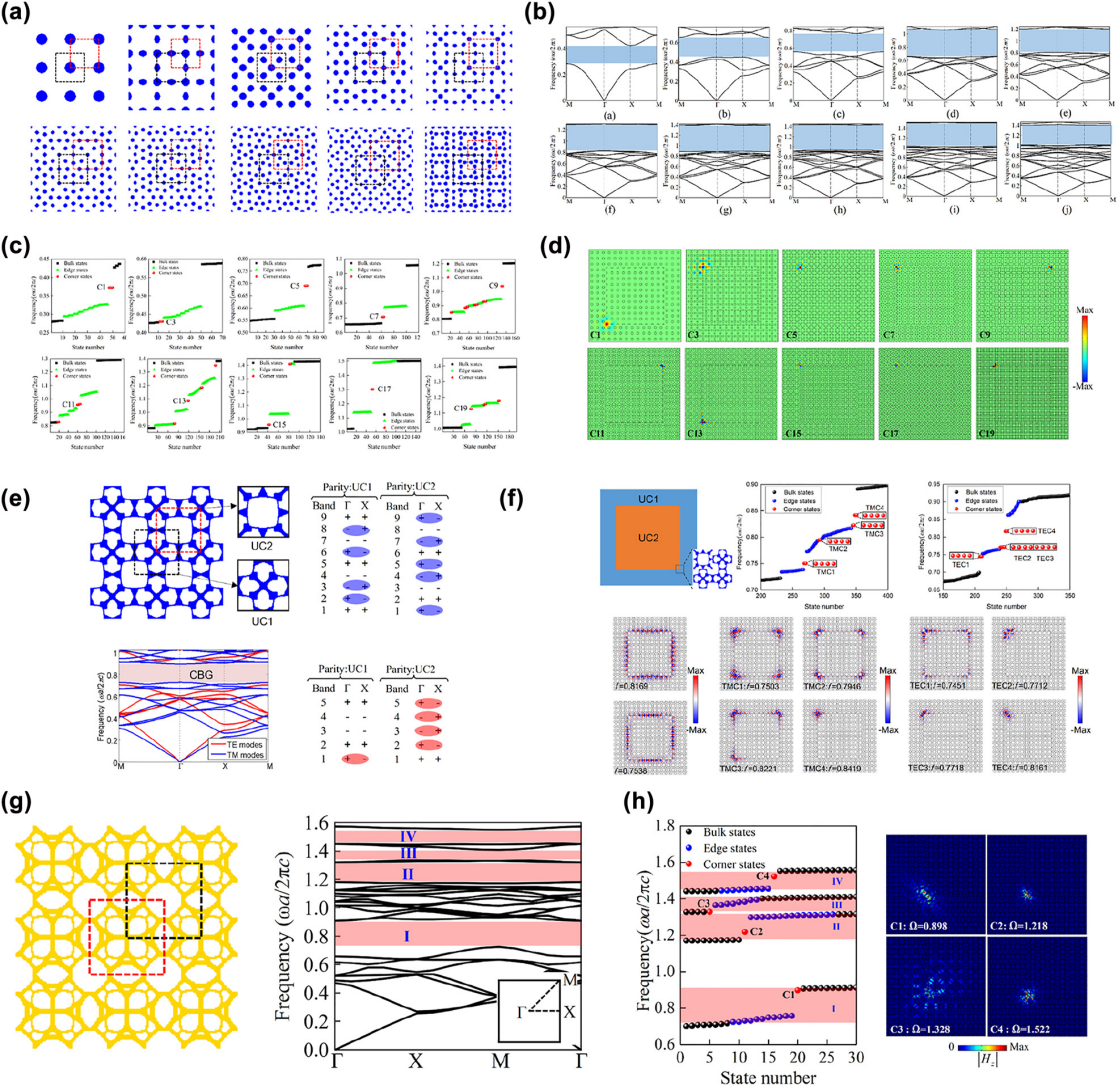
Inverse-designed second-order photonic TIs. (a) Optimized photonic crystals with odd-order bandgaps in Ref. [110]. (b) Band diagrams of optimized photonic crystals with odd-order bandgaps in Ref. [110]. (c) Calculated eigenfrequency spectrum of the second-order photonic TIs in Ref. [110]. (d) Eigenfield distributions of the corner states in Ref. [110]. (e) Optimized photonic crystal with dual-polarization bandgaps in Ref. [111]. (f) Observed dual-polarization corner states in Ref. [111]. (g) Optimized photonic crystal with four bandgaps in Ref. [112]. (h) Observed multiband corner states in Ref. [112].

The seminal second-order photonic TIs realized in C_4v_-symmetric lattices were based on the Su–Schrieffer–Heeger (SSH) model, whose lattice consists of four dielectric cylinders [25, 27]. The topological phase transition was realized by expanding or contracting the cylinders. Topological corner states were formed at corners of the metastructure consisting of nontrivial photonic crystals surrounded by trivial photonic crystals. However, the topological bandgap opened by this physics-inspired method was narrow, and as such the resulting corner states were less localized. To enlarge the topological bandgap to produce more localized corner states, Chen et al. [110] adopted the topology optimization method to design a series of second-order photonic TIs beyond the SSH model. Photonic crystals with C_4v_ symmetry were designed by maximizing the odd-order (from the first to the nineteenth) bandgaps via topology optimization (Figure 4(a) and (b)). Then topological trivial and nontrivial unit cells, denoted by the black and red boxes, were selected from the optimized photonic crystals in different ways; the nontrivial unit cells can be obtained by translating the trivial unit cells along the horizontal and vertical directions with half of the lattice constant simultaneously. As two choices of the unit cell from the photonic crystals with even-order bandgaps have the same topological feature, i.e., either all trivial or all nontrivial, thus cannot be used to construct an interface or corner hosting edge or corner states because one needs two domains having different topological features for these states to appear. Therefore, they only focus on exploring second-order topological phases with odd-order bandgaps. Tightly localized corner states appeared at corners of the metastructure made of nontrivial unit cells surrounded by trivial unit cells (Figure 4(c) and (d)), whose quality factors were significantly higher than those of corner states based on the SSH model.

Traditional second-order photonic TIs designed by physics-inspired methods only support corner states with one polarization, that is, either transverse magnetic (TM) mode or transverse electric (TE) mode. Chen et al. [111] designed a dual-polarization second-order photonic TI based on inverse design techniques. A photonic crystal with a complete bandgap for both TM and TE modes was designed by the topology optimization method (Figure 4(e)), where the orders of the complete bandgap for TM and TE modes are both odd. Then nontrivial and trivial unit cells were chosen from the optimized photonic crystals in two ways. At corners of metastructures made of nontrivial and trivial unit cells, highly localized corner states for both TM and TE modes were formed [Figure 4(f)]. The designed dual-polarization second-order photonic TI has potential applications in developing dual-polarization laser and interferometry with additional built-in topological protection.

Toward multiband applications of topological corner states in integrated optics, Chen et al. [112] created a second-order photonic TI supporting multiband corner states. A photonic crystal with four bandgaps was designed by the topology optimization method [Figure 4(g)], where the orders of these bandgaps are all odd. Likewise, nontrivial and trivial unit cells were chosen from the optimized photonic crystals in two ways [Figure 4(g)]. Four corner states within four bandgaps were observed at the corner of metastructres made of nontrivial and trivial unit cells [Figure 4(h)]. As the designed second-order TI was made of fully connected dielectric materials, it could be readily fabricated on nanoscale via electron beam lithography and integrated into on-chip circuits.

### 3.2 C_3_-symmetric lattices

**Figure 5:**
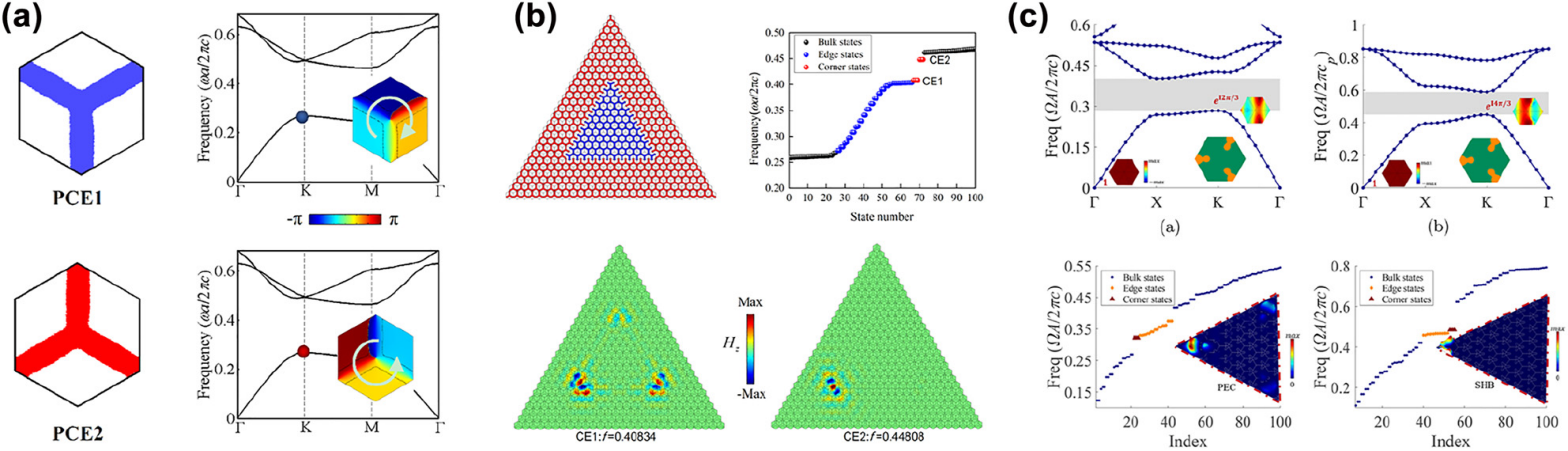
Inverse-designed second-order photonic and sonic TIs in C_3_-symmetric lattices. (a) Optimized C_3_ *-*symmetric photonic crystal and its inversion-symmetric partner in Ref. [115]. (b) Symmetric and antisymmetric corner states in the metastructure made of photonic crystals in (a) [115]. (c) Optimized gyromagnetic photonic crystal and sonic crystal (upper panel) and corner states in the metastructures made of the optimized structures (lower panel) in Ref. [117].

Recent works demonstrated that valley photonic/phononic crystals with C_3_ symmetry could be used to design second-order photonic/phononic TIs when the bulk bandgap was enlarged to induce an edge-state bandgap, within which corner states could appear [113, 114]. To open a large bulk bandgap, Chen et al. [115] adopted the topology optimization method to maximize the first-order bulk bandgap of a photonic crystal with C_3_ symmetry [PCE1 in Figure 5(a)]. Then an inversion-symmetric partner of the optimized photonic crystal was constructed to induce the topological phase transition [PCE2 in Figure 5(a)]. Highly localized symmetric and antisymmetric corner states were observed at corners of the metastructure made of PCE1s surrounded by PCE2s [Figure 5(b)]. Thereafter, Chen et al. [116] extended this topology optimization strategy to the design of C_3_ *-*symmetric sonic crystal for constructing second-order sonic TIs with tightly localized valley-selective corner states. Du et al. [117] proposed an explicit topology optimization-based design paradigm for the design of second-order photonic TIs (made of gyromagnetic materials) and second-order sonic TIs in C_3_ *-*symmetric and C_4v_ *-*symmetric lattices. Likewise, the optimization objective was to maximize the first-order bulk bandgap of the photonic/sonic crystal [upper panel in Figure 5(c)]. Highly localized corner states were observed at corners of the metastructure made of the optimized photonic/sonic crystal [lower panel in Figure 5(c)].

### 3.3 C_6v_-symmetric lattices

**Figure 6:**
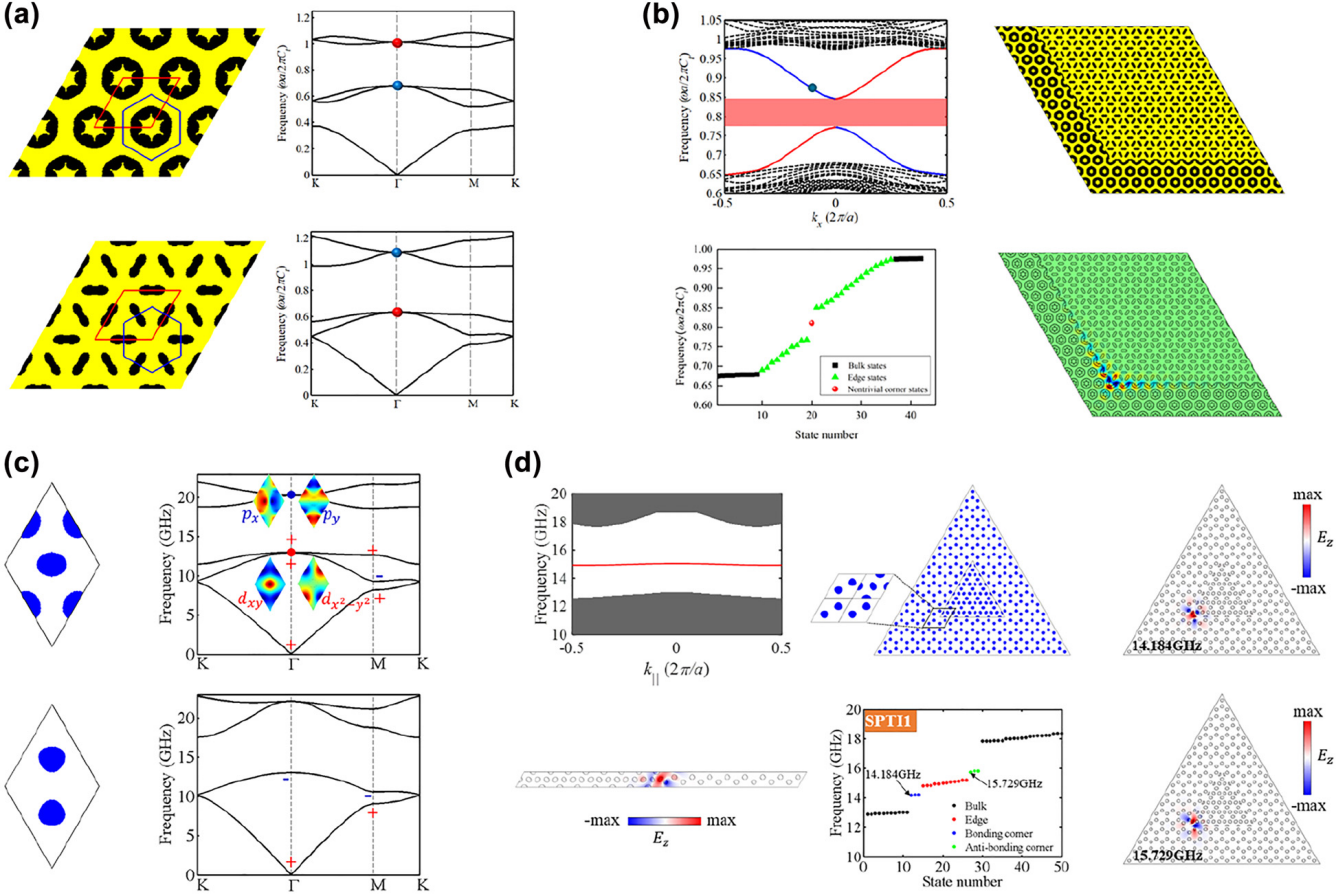
Inverse-designed second-order phononic and photonic TIs in C_6v_-symmetric lattices. (a) Optimized trivial and nontrivial phononic crystals in Ref. [118]. (b) Topological edge and corner states formed at the interface and corner of the second-order phononic TI in Ref. [118]. (c) Optimized trivial and nontrivial phononic crystals in Ref. [119]. (d) Topological edge and corner states formed at the interface and corner of the second-order photonic TI in Ref. [119].

With the development of high-order topology theory, researchers discovered that corner states could appear at the traditional QSHE-based photonic/phononic TIs when the edge states were gapped, which could be realized by further enlarging the overlapped bulk bandgap between trivial and nontrivial photonic/phononic crystals [23, 24]. Chen et al. [118] developed a topology optimization strategy to design nontrivial and trivial phononic crystals with C_6v_-symmetry for constructing second-order phononic TIs. Topology optimization therein aimed to simultaneously maximize the powers emitted by the artificially selected body forces to excite the *D* and *Q* modes at the target frequencies. Trivial (nontrivial) phononic crystal was created by exciting the *D* modes [blue balls in Figure 6(a)] below (above) the *Q* modes [red balls in Figure 6(a)]. The relative size of overlapped bandgap between trivial and nontrivial phononic crystals reached 36.14%, resulting in the gapped edge states [top left in Figure 6(b)]. Within the bandgap of edge states, highly localized corner states were formed at the corner of the metastructure made of trivial and nontrivial phononic crystals [Figure 6(b)]. Besides, the spatial decay of corner states was quantitatively characterized based on the complex band theory. To construct second-order photonic TIs in C_6v_-symmetric lattices, Chen et al. [119] designed a nontrivial photonic crystal by exciting the *D* modes above the *Q* modes [upper panel of Figure 6(c)] and a trivial photonic crystal by maximizing the second bandgap via topology optimization. An extra-wide overlapped bandgap with a relative size of 29.3% was reported. An almost flat edge state band was formed at the interface between trivial and nontrivial photonic crystals [left panel of Figure 6(d)]. Tightly localized symmetric and antisymmetric corner states were observed at corners of the metastructure made of nontrivial phononic crystals surrounded by trivial photonic crystals [middle and right panels of Figure 6(d)]. Additionally, by inversely designing several second-order photonic TIs with different frequencies for edge and corner states and purposely programming them, high-performance four-channel photonic routers for topological edge states and three-channel photonic routers for topological corner states were demonstrated.

## 4 Summary and prospective

In this review, we have summarized the recent development of using inverse design approaches to design photonic and phononic TIs, ranging from 1D TIs to 2D TIs based on QSHE and QVHE, and to high-order TIs in different symmetric lattices. It has been demonstrated that, after using advanced inverse design techniques, the performance of designed TIs could significantly exceed that of traditional TIs designed by physics-inspired methods. For example, the working bandwidth of topological edge states and the quality factor of topological corner states were significantly enhanced. More importantly, several previously unattainable functionalities were achieved, such as the second-order photonic TI hosting dual-polarization and multi-band corner states. Indeed, despite revolutionary developments have been made in the two independent research fields, i.e., photonic/phononic TIs and inverse design, the development of the interaction of these two fields are still at the infancy stage. We expect that this emerging field will promote future research along the following development directions.

– *Inverse design of 3D photonic and phononic TIs.* Prevailing works on the inverse design of photonic and phononic TIs currently are limited to the 1D and 2D systems, whereas inverse design of 3D photonic and phononic TIs remains unexplored. 3D photonic and phononic TIs possess richer topological phases and have more practical application values [120–124]. It is an interesting direction to exploit inverse design techniques to design various 3D photonic and phononic TIs with high performance, such as conventional 3D photonic and phononic TIs supporting gapless topological edge states with a wide working bandwidth and high-order 3D photonic and phononic TIs hosting highly localized hinge and corner states.
– *Inverse design of photonic and phononic TIs using deep learning.* Deep learning has already exhibited powerful abilities for designing novel phononic and photonic structures with extraordinary functions [75, 76]. However, using deep learning to design photonic and phononic TIs is still at its infancy stage. It is compelling to apply deep learning to design different kinds of photonic and phononic TIs with high performance, especially 2D and 3D photonic and phononic TIs.
– *Inverse design of photonic and phononic TIs based on new physics mechanisms.* With the development of topology theory, more and more new versions of photonic and phononic TIs have been proposed, such as non-Hermitian TIs [125, 126], nonlinear TIs [127, 128], Floquet TIs [129–132], Anderson TIs [133, 134], and so on. These TIs were mainly designed by the traditional physics-inspired trial-and-error methods, and as such their performance may be not optimal. Thus, it is desirable to adopt advanced inverse design techniques to design these new TIs to improve their performance.
– *Using inverse design techniques to discover new photonic and phononic topological phases.* Currently, inverse design techniques were mainly exploited to enhance the performance of photonic and phononic TIs based on well-known physics mechanisms. In the future, inverse design techniques including deep learning may not only facilitate the identification of new solid-state materials with previously overlooked topological properties but could also help to realize new photonic and phononic topological phases in the topological classes of materials from the Inorganic Crystal Structure Database (ICSD) [135–138] not explored previously in photonic and phononic contexts.
– *Inverse design of photonic and phononic TIs hosting multiband topological states.* Most of prevailing inverse-designed photonic and phononic TIs only support topological states within one bandgap. Toward multiband applications, it is desirable to design photonic and phononic TIs supporting multiband topological states. The work in Ref. [112] suggested a new route for creating multiple photonic corner states, which can be referred to engineer multiple high-order topological states in 2D and 3D photonic and phononic systems.
– *Inverse design of novel photonic and phononic topological devices.* One of the most important applications of photonic and phononic TIs is designing novel topological devices, such as topological photonic and phononic cavities [139, 140], topological photonic and phononic circuits [12, 141], and topological lasers [106, 142]. Their performance highly relies on the properties of the corresponding topological states, e.g., bandwidth, degree of localization and quality factor, which could be significantly improved by using inverse design techniques. We anticipate designing high-performance topological photonic and phononic devices based on inverse-designed photonic and phononic TIs.
